# A Dual-Labeling Probe for Super-Resolution Imaging to Detect Mitochondrial Reactive Sulfur Species in Live Cells

**DOI:** 10.3389/fphar.2022.871059

**Published:** 2022-06-01

**Authors:** Maomao Hu, Boyang Wang, Hongdan Zhang, Han Wang, Huixin Li, Xinyu Zhang, Jinjin Zhang, Qianrun Lu, Guiqian Fang, Juan Wang, Bo Dong

**Affiliations:** ^1^ Department of Cardiology, Shandong Provincial Hospital, Shandong University, Jinan, China; ^2^ Department of Cardiology, Shandong Provincial Hospital Affiliated to Shandong First Medical University, Jinan, China; ^3^ School of Life Sciences, Shandong First Medical University & Shandong Academy of Medical Sciences, Jinan, China; ^4^ Department of Cardiology, Shandong Traditional Chinese Medicine University, Jinan, China; ^5^ Shandong Cancer Hospital and Institute, Jinan, China; ^6^ Jinan Maternity and Child Care Hospital Affiliated to Shandong First Medical University, Jinan, China

**Keywords:** super-resolution imaging, mitochondria, RSS, small molecules probe, nanoscale

## Abstract

**Background:** Mitochondria are the main sites of reactive sulfur species (RSS) production in living cells. RSS in mitochondria play an important role in physiological and pathological processes of life. In this study, a dual-labeling probe that could simultaneously label the mitochondrial membrane and matrix was designed to quantitatively detect RSS of mitochondria in living cells using nano-level super-resolution imaging.

**Methods:** A fluorescent probe CPE was designed and synthesized. The cytotoxicity of CPE was determined and co-localization of CPE with a commercial mitochondrial probe was analyzed in HeLa cells. Then, the uptake patterns of CPE in HeLa cells at different temperatures and endocytosis levels were investigated. The staining characteristics of CPE under different conditions were imaged and quantitated under structured illumination microscopy.

**Results:** A fluorescence probe CPE reacting to RSS was developed, which could simultaneously label the mitochondrial membrane with green fluorescence and the mitochondrial matrix with red fluorescence. CPE was able to demonstrate the mitochondrial morphology and detect the changes of RSS in mitochondria. With the increase of mitochondrial RSS concentration, the light of the red matrix will be quenched.

**Conclusion:** CPE provides a strategy for the design of probes and an attractive tool for accurate examination to changes of mitochondrial morphology and RSS in mitochondria in living cells at the nanoscale.

## 1 Introduction

Mitochondria serve as very important organelles in eukaryotic cells, which mainly provide energy for cell activities and are known as the “power factory” of cells (Yousif et al., 2009; Rezaeian et al., 2022; Chen et al., 2021). In addition, mitochondria have various functions of other vital life activities including participating in lipid synthesis, buffering intracellular calcium, and modulating immune response. Moreover, mitochondria are the main sites of reactive sulfur species (RSS) production in living organisms (Vinten, 2020). Intracellular RSS have emerged as a general term for active sulfur-containing biomolecules including hydrogen sulfide (H_2_S), sulfur dioxide (SO_2_), cysteine (Cys), homocysteine (Hcy), and reduced glutathione (GSH) that play an important role in many physiological and pathological processes. For example, sulfur dioxide (SO_2_) is not only likely a primary energy generation for important biosynthetic reactions but also involved in a multitude of biological signaling (Lau and Pluth, 2019). Moderate concentrations of RSS can be healthy, but many studies have shown that excessive RSS would be associated with many diseases, including cardiovascular diseases, neurological diseases, and tumors (Cai et al., 2010; Yan et al., 2019; Shi et al., 2022). Therefore, it is of great significance to develop fluorescent probes that can rapidly, real-time, and accurately detect changes of RSS concentrations in living cells for the diagnosis of related diseases.

At present, fluorescent probes have been developed to detect the content of active sulfur in mitochondria, with good selectivity, quick response (3 min), low cytotoxicity, and good cell permeability (Bai et al., 2021). However, they are not able to reflect the morphology of mitochondria, which could be damaged by excess active sulfur in the body. Mitochondrial morphology including the integrity of mitochondrial outer membrane and the presence of cristae is the most direct reflection of mitochondrial functional integrity (Wiemerslage and Lee, 2016; Ke et al., 2018). In addition, morphological changes in mitochondria that were divided into mitochondrial swelling, rupture, integrity of inner or outer membrane destructions, and mitochondrial crest fracture play a crucial role in occurrence and development of mitochondria-related diseases (Alirol and Martinou, 2006; Yu et al., 2006; Shao et al., 2020). Apart from the deficiencies mentioned earlier, due to the limited resolution and sensitivity of conventional electron microscopy, mitochondria cannot be clearly distinguished from other membranous structures, which is sometimes confusing (Chen et al., 2019).

To solve this problem, we developed a small-molecule fluorescent probe containing nitrogen ions with specific organelle-targeting ability of the mitochondrial membrane and matrix, which could not only demonstrate the morphology of mitochondria but also tract the changes of RSS in the matrix of mitochondria. At the same time, recent development of the extended-resolution microscopy technique and structured illumination microscopy (SIM) (Gustafsson, 2000; Huang et al., 2018) have made it possible to investigate delicate structures of mitochondria in living cells at the nanoscale level (Chen et al., 2018), and based on that, we incubated HeLa cells with **CPE** for 1 h, and then imaged under SIM using a dual-channel mode with excitation at 405 and 561 nm. As expected, **CPE** labeled the mitochondria membrane with green fluorescence and the matrix with red fluorescence. With the increase of active sulfur in the mitochondria, the red matrix fluorescence would be quenched. Meanwhile, mitochondrial morphology may also be changed, which make it possible to further clarify the relationship between the content of active sulfur in mitochondria and the functional status of mitochondria at the nanoscale level. According to the aforementioned information, **CPE** may be a new tool for tracking RSS and the function of mitochondria under SIM, providing a powerful method for investigating diagnosis and treatment strategies for mitochondria-related diseases.

## 2 Results and Discussion

### 2.1 Characterization of CPE

**FIGURE 1:**
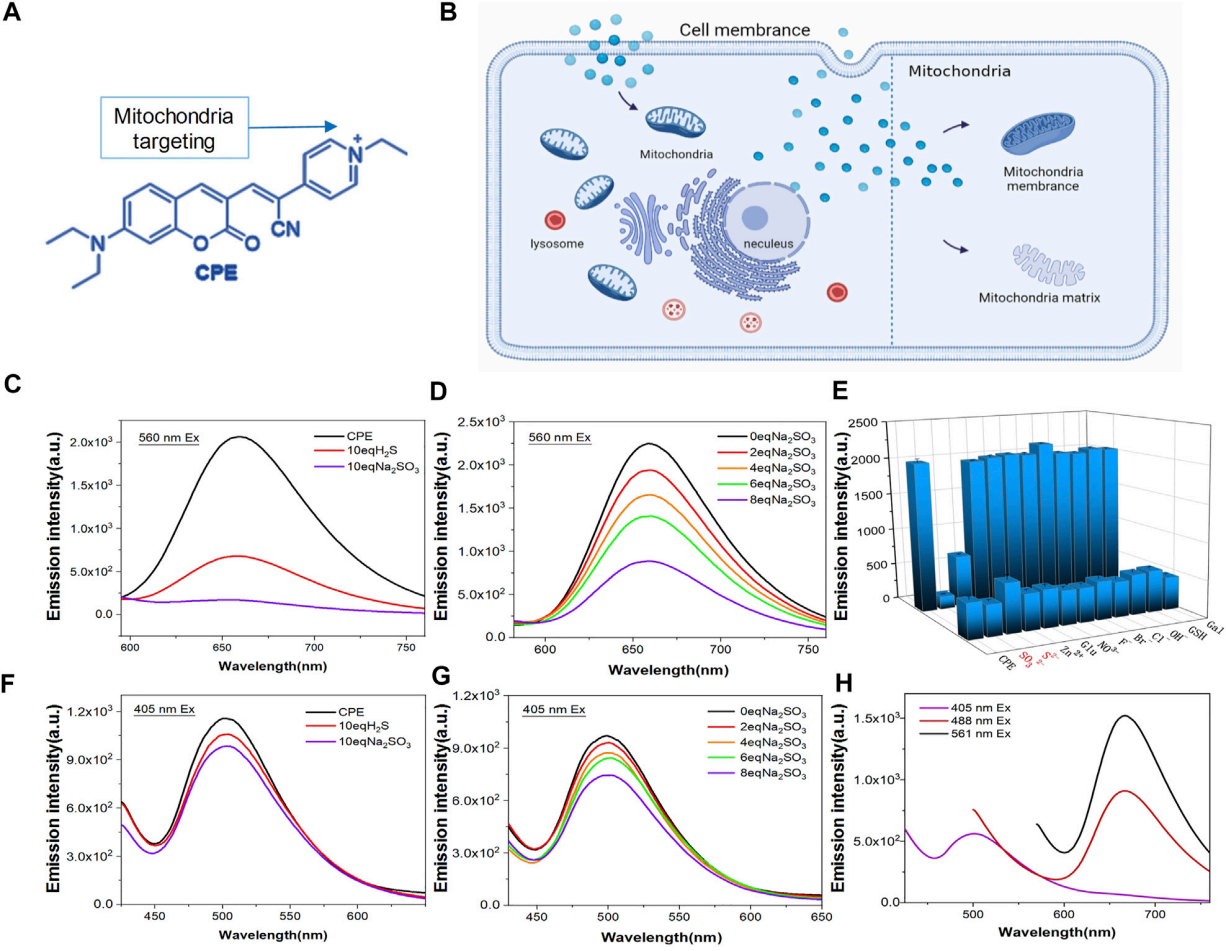
Design and fluorescence characterization of CPE. **(A)** Chemical structure of CPE. **(B)** Staining organelles of CPE. **(E)** Responsive substance of CPE. **(H)** CPE emission spectrogram. **(C,F)** Fluorescence spectra determined with H_2_S and Na_2_SO_3_ treatments. **(D,G)** Fluorescence spectra were determined after response with different concentrations of Na_2_SO_3_.

Mitochondria are two-membrane-bound sub-organelles surrounded by an outer and an inner smooth membrane, which is folded to form the cristae (Sasaki, 2010). The inner mitochondrial membrane encircles a space identified as the matrix. The membrane potential difference of mitochondria tends to attract positively charged molecules to accumulate in its interior (Leung et al., 2013). Based on this, we modified the coumarin group with pyridine to make it positively charged. Under the attraction of mitochondrial membrane potential difference, **CPE** can accurately target mitochondria (Figures 1A,B) (Zhao et al., 2013; Zhao et al., 2014; Zhao et al., 2015; Shi et al., 2016; Gui et al., 2017). In addition, the fluorophores possess two emission peaks (∼500 and 660 nm) (Figure 1H), which provides them the chance to label the mitochondrial membrane and matrix. To indicate this point, we incubated **CPE** *in vitro* with lecithin for 1 h, and then imaged using SIM with 405 and 561 channels emitted. As expected, lecithin that loaded **CPE** showed green fluorescence at the excitation of 405 nm and red fluorescence at the excitation of 561 nm (Supplementary Figure S1), which suggests that **CPE** is a double-labeled probe. In addition, the fluorophores can react with RSS such as H_2_S and SO_2_ (Figures 1C,F). It is shown that the color of **CPE** changed from mauve to light yellow when it reacted with Na_2_SO_3_ *in vitro* experiments (Supplementary Figure S2). To measure the degree of response to RSS, we used Na_2_S as an H_2_S donor and Na_2_SO_3_ as an SO_2_ donor to simulate the environment rich in RSS of mitochondria. As observed, the fluorescence of **CPE** decreased distinctly with the increase of RSS (Figures 1D,G). The 561 excited light was quenched at 10 equivalent Na_2_SO_3_, while the 405 excited light did not quench and remained at a certain fluorescence intensity still. Moreover, the probe did not respond to other biologically relevant species such as H_2_O_2_, ClO^−^, and F^−^ (Figure 1E). These results suggest that **CPE** can not only respond to active sulfur and detect the active sulfur content but also have the potential to label mitochondria.

### 2.2 Imaging of CPE in Living Cells

**FIGURE 2:**
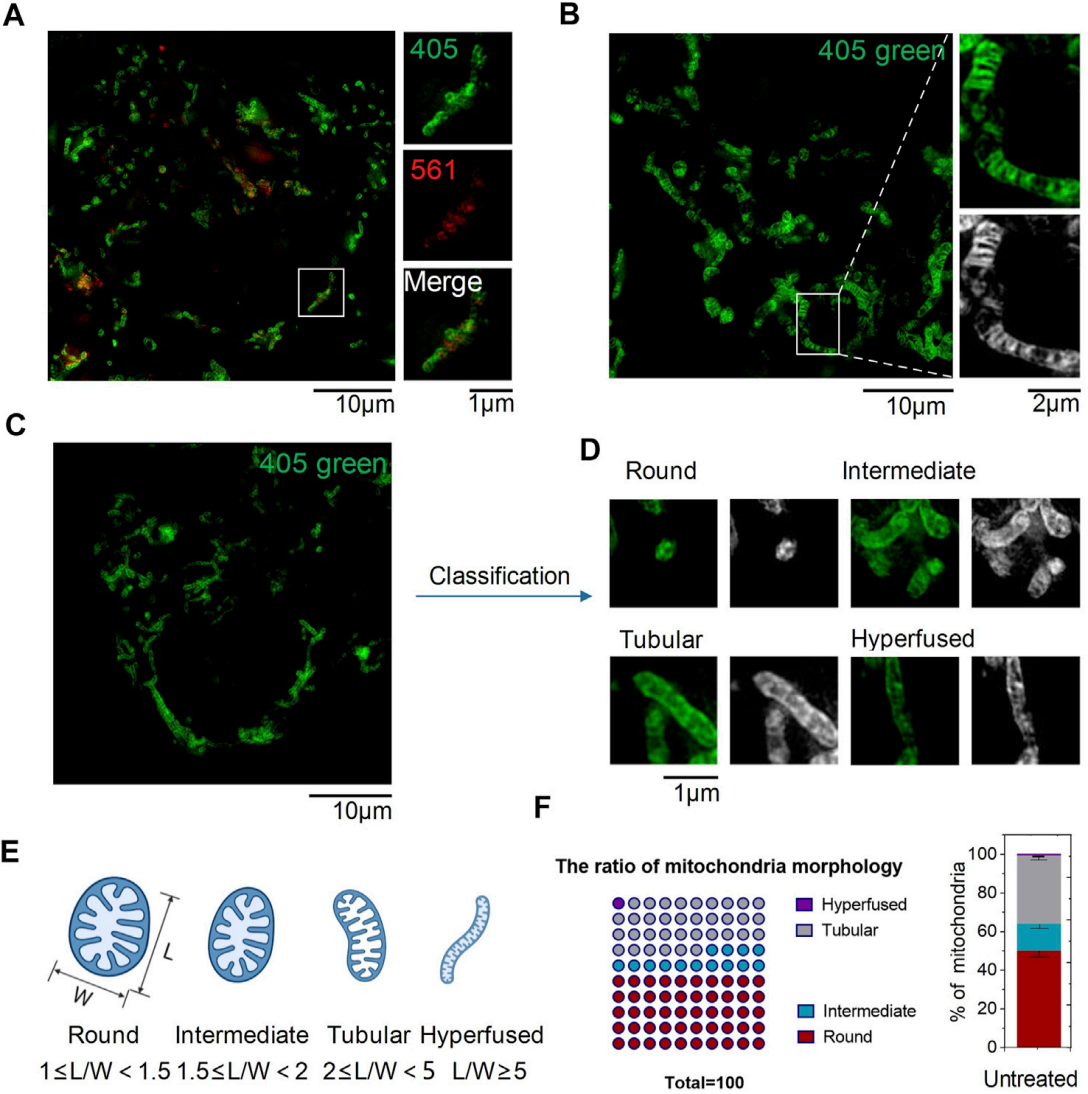
SIM images of CPE puncta in HeLa cells (λ_ex_1 = 405 nm and λ_ex_2 = 561 nm). **(A)** SIM image of cells labeled with CPE (10 µM) for 1 h. **(B)** Mitochondrial crista. **(C–E)** Mitochondrial morphology of distribution parameters of *L/W*. **(F)** Ratio of mitochondrial morphology.

To verify whether **CPE** could target organelles in living cells or not, HeLa cells were incubated with **CPE** and imaged under SIM using a dual-channel mode with excitation at 405 and 561 nm. As shown in SIM images, green fluorescence excited at 405 nm stained the outer membrane of mitochondria, which revealed fibrous, rod-like, and punctate morphology (Figures 2C,D), consistent with previous literature reports (Sasaki, 2010). Red particles or fibers with weaker fluorescence excited at 561 were encased in a green membrane, illustrating it targets the mitochondrial matrix (Figure 2A). Here is the surprise, **CPE** could show the crest line distribution of mitochondria (Figure 2B), which is closely associated with the pathology of cancer, osteoarthritis, and AIDS (Guarani et al., 2015; Blanco et al., 2011). In addition, this scene in live cells could only be captured by SIM compared to other reported methods of imaging (Shao et al., 2020). Therefore, the combination of SIM can take advantage of the probe **CPE**, which suggests its potential for use in the diagnosis of mitochondrial diseases. Next, we used the length-to-width ratio (*L*/*W*) to quantitatively analyze the distribution of mitochondria and found that various morphologies could be assigned into four groups as follows: hyperfused (*L*/*W* ≥ 5.0), tubular (2.0 ≤ *L*/*W* < 5.0), intermediate (1.5 ≤ *L*/*W* < 2.0), and round or nearly round (1.0 ≤ *L*/*W* < 1.5) (Figures 2C–E) (Cao et al., 2017). The fibrous, rod-shaped, and spotted once mentioned earlier might be classified as hyperfused, tubular, and round (Figure 2F), and then, we used CCK-8 assay to evaluate the cytotoxicity of **CPE** to HeLa cells (Qin et al., 2015). No cytotoxicity was shown at the range of 0–20 μM to HeLa cells during 24 h, demonstrating that 10 μM is a relatively safe working concentration for **CPE** with no interference in mitochondrial imaging under SIM in living cells. Finally, different temperatures and endocytosis levels of **CPE** incubated to HeLa cells were observed to define the uptake properties of **CPE**. The cells showed weaker fluorescence when incubated with **CPE** at 4°C (Supplementary Figure S3) or with an endocytosis inhibitor (NH_4_Cl) (Supplementary Figure S4) than that of cells incubated with **CPE** at 37°C (Fang et al., 2019), whether it is red or green fluorescence. These results strongly indicate that **CPE** enters cells through energy-dependent endocytosis. Therefore, we conclude that **CPE** stains organelles in living cells with low toxicity and good cell permeability.

### 2.3 CPE Could Specifically Label Mitochondria

**FIGURE 3:**
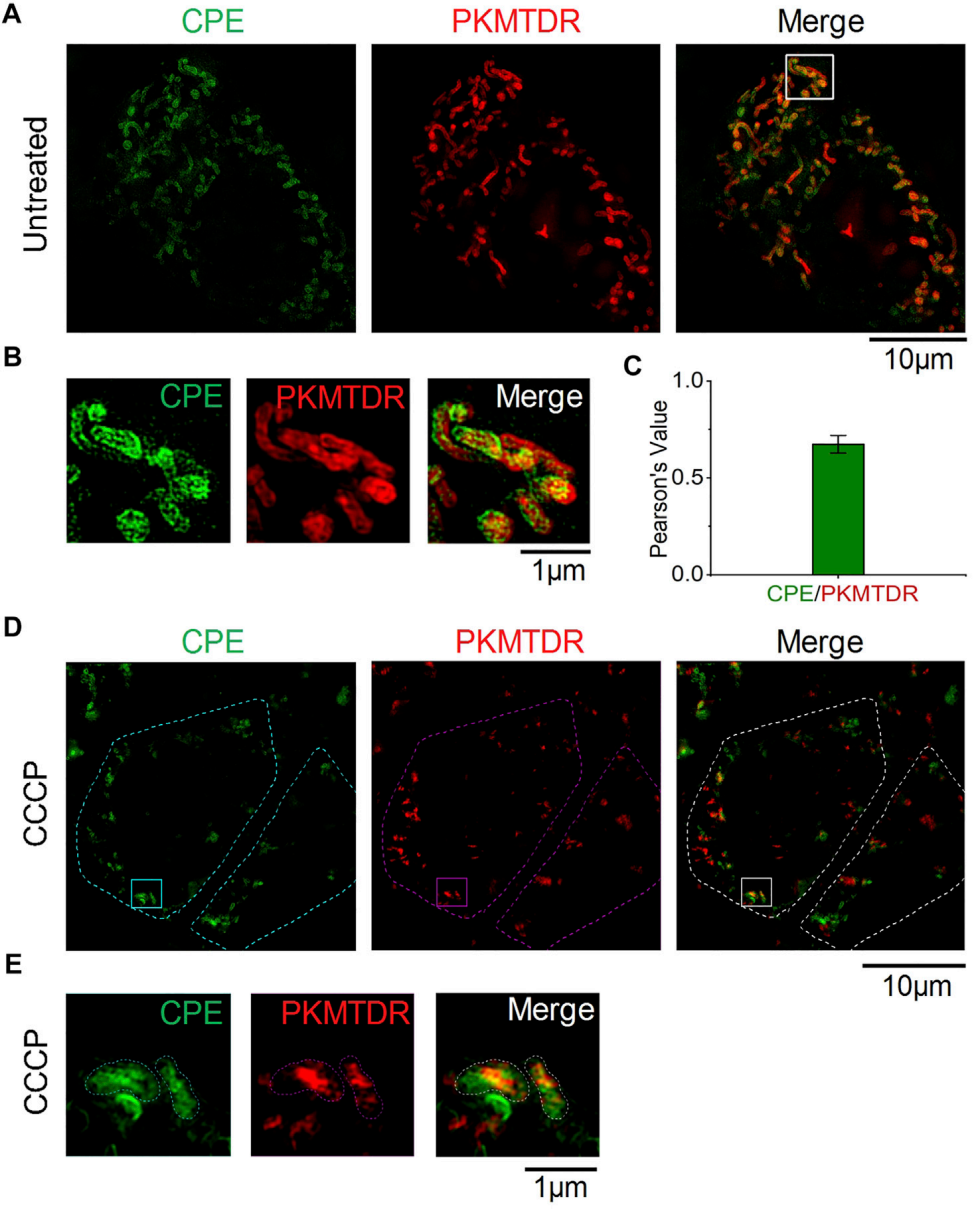
Colocalization of CPE and PKMTDR in HeLa cells under SIM. **(A)** Merged SIM images of cells stained with CPE and PKMTDR. **(B)** Enlarged images of the indicated regions in **(A)**. **(C)** Quantitative analysis of the colocalization between CPE and PKMTDR. **(D)** CCCP-treated HeLa cells. **(E)** Enlarged images of the indicated regions in **(D)**

To determine whether **CPE** can specifically label mitochondria, we co-stained the cells with a commercial probe, PKMTDR, for 1 h. The following merged SIM images revealed that the green fluorescence signal of **CPE** colocalized well with the red fluorescence signal of PKMTDR (Figures 3A,B), whose Pearson colocalization coefficient (PCC) was as high as 0.72 with PKMTDR (Figure 3C). It is clear that **CPE** has high specificity for mitochondrial attachment. Next, we observed whether **CPE-**labeled mitochondria depends on mitochondrial membrane potential (MMP) or not. To damage the membrane potential of mitochondria, HeLa cells were treated with 10 μM carbonyl cyanide m-chlorophenyl hydrazone (CCCP), which was used as a common mitophagy inducer (Chen et al., 2020a). After that, we re-stained the cells with both **CPE** and PKMTDR for co-location imaging (Figures 3D,E), while most did not attach to broken mitochondria. This indicates that labeled mitochondria depends on MMP. These results show that **CPE** could not only label mitochondria specifically but also provide references for measuring MMP.

### 2.4 CPE Can Detect the Active Sulfur Content and Indicate Mitochondrial Status

**FIGURE 4:**
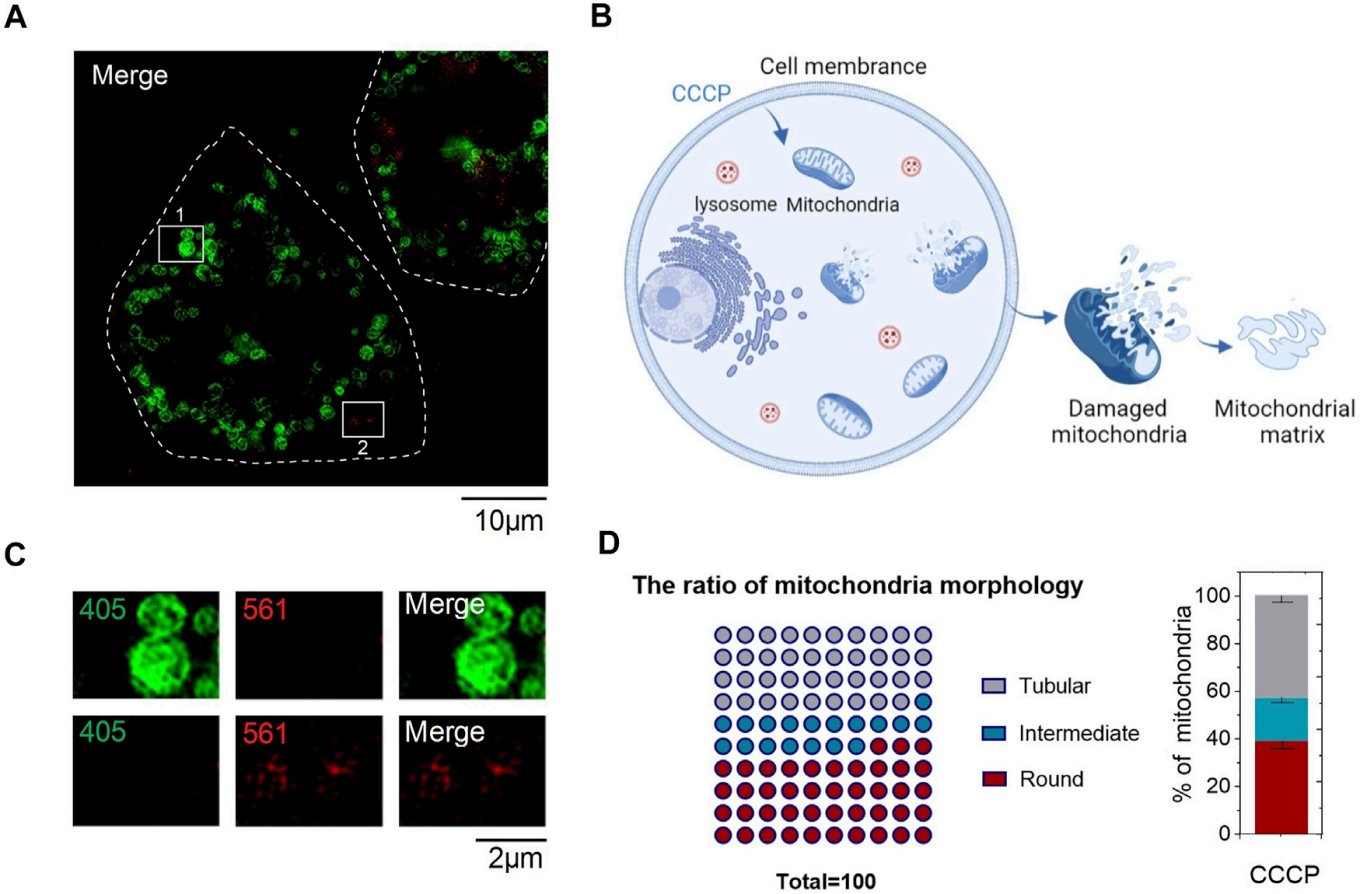
Staining characteristics of CPE in CCCP-treated HeLa cells. **(A)** CCCP-treated HeLa cells. **(B)** Sketch map of staining characteristics of CPE in HeLa cells after CCCP treatment. **(C)** Enlarged images of the indicated regions in **(A)**. **(D)** Quantitative analysis for mitochondrial morphology of HeLa cells treated after CCCP.

To confirm whether **CPE** can detect the content of active sulfur in mitochondria, we processed mitochondria with CCCP of 50 μM for 30 min. Mitochondria were broken into round shapes (Figure 4A), consistent with previous literature reports (Chen et al., 2020b). Then, *L*/*W* was used to quantitatively analyze the distribution of mitochondrial morphology as before. After that, we used automatic analysis software (ImageJ) to calculate the distribution of individual mitochondria in CCCP-treated HeLa cells and found that the ratio of round structures accounts for nearly a half (Figure 4D), which indicates the mitochondria were in an unhealthy status. In addition, we found that red fluorescent excited by 561 was quenched in round shape mitochondria (Figure 4C), which was due to CCCP treatment increased the concentrations of ROS (Kane et al., 2018), high concentrations of ROS then increased RSS levels (Tabassum and Jeong, 2019). However, there was still some red fluorescent outside mitochondria, thus we hypothesized that it was caused by the destruction of mitochondrial outer membrane and the outflow of mitochondrial matrix (Figure 4B). Meanwhile, the content of active sulfur flowing out of the matrix was not enough to quench red fluorescent. All these indicate that **CPE** has the potential of detecting active sulfur in mitochondria and judging the status of mitochondria, which can provide a powerful reference value for the diagnosis of mitochondrial diseases.

## 3 Conclusion

Mitochondria-related diseases are closely associated to mitochondrial damage, which is characterized by morphological distribution changes and crest damage (Schapira, 2006; Senyilmaz et al., 2015). In addition, RSS in mitochondria can regulate mitochondrial morphogenesis and play a crucial role in the physiological and pathological processes of living organisms (Kashatus, 2018). However, traditional methods of observing mitochondrial morphology, such as transmission electron microscopy, magnetic resonance imaging, and confocal fluorescence microscopy, are unable to capture the morphology of living cells and simulate the actual *in vivo* state (Chen et al., 2019). To address this problem, here, we developed **CPE**, a dual-labeling probe enabling the evaluation for mitochondrial morphology and the detection of RSS through simultaneous labeling of the mitochondria membrane and matrix in living cells, which make it possible for the diagnosis of early mitochondria-related diseases under SIM. Thus, **CPE** not only provides strategies for the design of accurate positioning probes but may also become a powerful approach for investigating mitochondrial biology.

## 4 Experimental Sections

### 4.1 Synthetic Route

A mixture of 7-(diethylaMino)-2-oxo-2H-chromene-3-carbaldehyde (0.26 g, 1 mmol) and 4-pyridineacetonitrile (0.12 g, 1 mmol) refluxed in dry ethanol (15 ml). A large amount of brown powder solids precipitated after reacted overnight. After cooling to room temperature, the crude product was filtered and washed with cool acetonitrile. Subsequently, a mixture of the previous product (0.172 g, 0.5 mmol) and iodoethane (0.47 g, 3 mmol) refluxed in acetonitrile (3 ml). A large amount of purple powder solids precipitated after reacted for 24 h. Then, the crude product was obtained by filtering, followed by washing with cold ethanol and ethyl ester. A purple compound **CPE** (0.11 g, 0.3 mmol, 60%) was obtained after drying. ^1^H NMR (600 MHz, DMSO-*d*_*6*_) *δ* (ppm) (Supplementary Figure S5): 9.05 (d, *J* = 7.1 Hz, 2H), 8.84 (s, 1H), 8.39 (s, 1H), 8.32 (d, *J* = 7.1 Hz, 2H), 7.66 (d, *J* = 9.1 Hz, 1H), 6.88 (dd, *J* = 9.1, 2.4 Hz, 1H), 6.68 (d, *J* = 2.2 Hz, 1H), 4.62 (q, *J* = 7.3 Hz, 2H), 3.56 (q, *J* = 7.0 Hz, 4H), 1.55 (t, *J* = 7.3 Hz, 3H), and 1.18 (t, *J* = 7.1 Hz, 6H). ^13^C NMR (151 MHz, DMSO-*d*_*6*_) *δ* (ppm) (Supplementary Figure S6): 160.46, 158.10, 154.22, 150.01, 145.28, 144.98, 144.82, 132.97, 123.23 116.91, 111.58, 111.31, 108.85, 102.17, 97.29, 56.04, 45.28, 16.61, and 12.94. HRMS *m/z* (Supplementary Figure S7): calculated for C_23_H_24_N_3_O_2_^+^ [M]^+^: 374.1863, found 374.1775.

### 4.2 General Materials

Dulbecco’s modified Eagle’s medium (#11965118, DMEM), phenol-free medium (#1894117), penicillin–streptomycin (#15140163, 10,000 units/ml), trypsin-EDTA (#25200–072), and other reagents for cell culture were obtained from Gibco BRL (Grand Island, NY, United States). Fetal bovine serum (FBS) was obtained from VivaCell (Shanghai, China). HeLa cells were gifted from the Chunyan Liu’s lab (Shandong First Medical University).

### 4.3 Cell Culture

HeLa cells were cultured in Dulbecco modified Eagle medium supplemented with 10% fetal bovine serum, penicillin (100 μg/ml), and streptomycin (100 μg/ml) in a 5% CO_2_ humidified incubator at 37°C.

### 4.4 Experiments *In Vitro*

**CPE** (10 μM) was put into a color dish and allowed to react with different concentrations such as 0, 2, 4, 6, and 8 eq., Na_2_SO_2_ or H_2_S in order to complete the reactive sulfur concentration response experiment, and then the fluorescence spectra of **CPE** at different concentrations were detected. Next, we incubated **CPE** with lecithin for 1 h and imaged under SIM to search for the luminescence properties of **CPE**.

### 4.5 Cell Culture and Imaging Under OMX 3D-SIM

HeLa cells were seeded on 35 mm glass-bottom micro dishes at a density of 1×10^5^ and incubated with 2 ml of DMEM medium supplemented with 10% FBS for 24 h. After that, cells were incubated with 10 μM **CPE** for 1 h and washed with fresh DMEM for five times. At last, the cells with no phenol in culture medium were imaged under an OMX 3D-SIM extended-resolution microscope. Images were acquired at 512 × 512, with a step size of 0.125 μm, and **CPE** was excited at 405 and 561 nm.

### 4.6 Cytotoxicity Assay

The Cell Counting Kit-8 (CCK-8) assay was used to measure the cytotoxicity assay. HeLa cells at a density of 8×10^3^ every well were seeded in a 96-well plate in DMEM with 10% FBS at 37°C for 24 h. Then, the original medium was replaced with 100 μL fresh medium, each well containing **CPE** with the concentrations of 0, 1, 5, 10, and 20 μM. After 24 h incubation, 10 μL CCK-8 solution was added to each well, and the plate was incubated in the incubator for 1 h. Finally, the absorbance at 450 nm was determined by enzyme-linked immunosorbent assay.

### 4.7 Colocalization Experiments

Cells at a density of 1×10^5^ were seeded on 35 mm glass-bottom culture dishes and incubated with 2 ml of DMEM medium supplemented with 10% FBS. After 24 h incubation, cells were incubated with 100 nM PKMTDR and 10 μM **CPE** for 1 h. Finally, the cells were cultured in a phenol-free medium and imaged under an OMX 3D-SIM. PKMTDR was excited at 561 nm, and **CPE** was at 405 nm. The images were analyzed using ImageJ.

### 4.8 Cellular Uptake Assay

HeLa cells were stained with 10 μM **CPE** under different conditions. 37 °C: the cells were stained with **CPE** at 37 °C for 1 h. 4 °C: the cells were stained with **CPE** at 4 °C for 1 h. NH_4_Cl: the cells were pre-incubated with NH_4_Cl (50 mM) in FBS-free DMEM at 37 °C for 2 h, and then incubated with **CPE** at 37 °C for 1 h.

### 4.9 Statistical Analysis

Statistical analysis was performed with Prism 9 (GraphPad) and ImageJ. Statistical significances and sample sizes in all graphs are indicated in the corresponding figure legends.

## Data Availability

The original contributions presented in the study are included in the article/Supplementary Material, further inquiries can be directed to the corresponding authors.

## Abbreviations

SIM, structured illumination microscopy; CCK-8, Cell Counting Kit-8; TEM, traditional transmission microscope; H_2_S, hydrogen sulfide; SO_2_, sulfur dioxide; Cys, cysteine; Hcy, homocysteine; GSH, reduced glutathione; RSS, reactive sulfur species; MMP, mitochondrial membrane potential.

## References

[B1] Alirol E. Martinou J. C. (2006). Mitochondria and Cancer: Is There a Morphological Connection? Oncogene 25 (34), 4706–4716. 10.1038/sj.onc.1209600 PubMed Abstract | 10.1038/sj.onc.1209600 | Google Scholar 16892084

[B2] Bai H. Liu H. Chen X. Hu R. Li M. He W. (2021). Augmenting Photosynthesis through Facile AIEgen-Chloroplast Conjugation and Efficient Solar Energy Utilization. Mater. Horiz. 8 (5), 1433–1438. 10.1039/d1mh00012h PubMed Abstract | 10.1039/d1mh00012h | Google Scholar 34846450

[B3] Blanco F. J. Rego I. Ruiz-Romero C. (2011). The Role of Mitochondria in Osteoarthritis. Nat. Rev. Rheumatol. 7 (3), 161–169. 10.1038/nrrheum.2010.213 PubMed Abstract | 10.1038/nrrheum.2010.213 | Google Scholar 21200395

[B4] Cai W. J. Wang M. J. Ju L. H. Wang C. Zhu Y. C. (2010). Hydrogen Sulfide Induces Human colon Cancer Cell Proliferation: Role of Akt, ERK and P21. Cell. Biol. Int. 34 (6), 565–572. 10.1042/CBI20090368 PubMed Abstract | 10.1042/CBI20090368 | Google Scholar 20184555

[B5] Cao X. Wang H. Wang Z. Wang Q. Zhang S. Deng Y. (2017). *In Vivo* imaging Reveals Mitophagy independence in the Maintenance of Axonal Mitochondria during normal Aging. Aging Cell 16 (5), 1180–1190. 10.1111/acel.12654 PubMed Abstract | 10.1111/acel.12654 | Google Scholar 28782874 PMC5595681

[B6] Chen H. Wang H. Wei Y. Hu M. Dong B. Fang H. (2021). Super-resolution Imaging Reveals the Subcellular Distribution of Dextran at the Nanoscale in Living Cells. Chin. Chem. Lett. 10.1016/j.cclet.2021.10.025 10.1016/j.cclet.2021.10.025 | Google Scholar

[B7] Chen Q. Fang H. Shao X. Tian Z. Geng S. Zhang Y. (2020a). A Dual-Labeling Probe to Track Functional Mitochondria-Lysosome Interactions in Live Cells. Nat. Commun. 11 (1), 6290. 10.1038/s41467-020-20067-6 PubMed Abstract | 10.1038/s41467-020-20067-6 | Google Scholar 33293545 PMC7722883

[B8] Chen Q. Jin C. Shao X. Guan R. Tian Z. Wang C. (2018). Super-Resolution Tracking of Mitochondrial Dynamics with an Iridium(III) Luminophore. Small 14 (41), e1802166. 10.1002/smll.201802166 PubMed Abstract | 10.1002/smll.201802166 | Google Scholar 30350549

[B9] Chen Q. Shao X. Hao M. Fang H. Guan R. Tian Z. (2020b). Quantitative Analysis of Interactive Behavior of Mitochondria and Lysosomes Using Structured Illumination Microscopy. Biomaterials 250, 120059. 10.1016/j.biomaterials.2020.120059 PubMed Abstract | 10.1016/j.biomaterials.2020.120059 | Google Scholar 32339858 PMC7236803

[B10] Chen Q. Shao X. Tian Z. Chen Y. Mondal P. Liu F. (2019). Nanoscale Monitoring of Mitochondria and Lysosome Interactions for Drug Screening and Discovery. Nano Res. 12, 1009–1015. 10.1007/s12274-019-2331-x 10.1007/s12274-019-2331-x | Google Scholar

[B11] Fang H. Yao S. Chen Q. Liu C. Cai Y. Geng S. (2019). *De Novo*-Designed Near-Infrared Nanoaggregates for Super-resolution Monitoring of Lysosomes in Cells, in Whole Organoids, and *In Vivo* . ACS. Nano 13 (12), 14426–14436. 10.1021/acsnano.9b08011 PubMed Abstract | 10.1021/acsnano.9b08011 | Google Scholar 31799834 PMC7255917

[B12] Guarani V. McNeill E. M. Paulo J. A. Huttlin E. L. Fröhlich F. Gygi S. P. (2015). QIL1 Is a Novel Mitochondrial Protein Required for MICOS Complex Stability and Cristae Morphology. Elife 4, e06265. 10.7554/eLife.06265 10.7554/eLife.06265 | Google Scholar PMC4439739 25997101

[B13] Gui C. Zhao E. Kwok R. T. K. Leung A. C. S. Lam J. W. Y. Jiang M. (2017). AIE-active Theranostic System: Selective Staining and Killing of Cancer Cells. Chem. Sci. 8 (3), 1822–1830. 10.1039/c6sc04947h PubMed Abstract | 10.1039/c6sc04947h | Google Scholar 30155198 PMC6092713

[B14] Gustafsson M. G. (2000). Surpassing the Lateral Resolution Limit by a Factor of Two Using Structured Illumination Microscopy. J. Microsc. 198 (Pt2), 82–87. 10.1046/j.1365-2818.2000.00710.x PubMed Abstract | 10.1046/j.1365-2818.2000.00710.x | Google Scholar 10810003

[B15] Huang X. Fan J. Li L. Liu H. Wu R. Wu Y. (2018). Fast, Long-Term, Super-resolution Imaging with Hessian Structured Illumination Microscopy. Nat. Biotechnol. 36 (5), 451–459. 10.1038/nbt.4115 PubMed Abstract | 10.1038/nbt.4115 | Google Scholar 29644998

[B16] Kane M. S. Paris A. Codron P. Cassereau J. Procaccio V. Lenaers G. (2018). Current Mechanistic Insights into the CCCP-Induced Cell Survival Response. Biochem. Pharmacol. 148, 100–110. 10.1016/j.bcp.2017.12.018 PubMed Abstract | 10.1016/j.bcp.2017.12.018 | Google Scholar 29277693

[B17] Kashatus D. F. (2018). The Regulation of Tumor Cell Physiology by Mitochondrial Dynamics. Biochem. Biophys. Res. Commun. 500 (1), 9–16. 10.1016/j.bbrc.2017.06.192 PubMed Abstract | 10.1016/j.bbrc.2017.06.192 | Google Scholar 28676396 PMC5748380

[B18] Ke H. Dass S. Morrisey J. M. Mather M. W. Vaidya A. B. (2018). The Mitochondrial Ribosomal Protein L13 Is Critical for the Structural and Functional Integrity of the Mitochondrion in Plasmodium Falciparum. J. Biol. Chem. 293 (21), 8128–8137. 10.1074/jbc.RA118.002552 PubMed Abstract | 10.1074/jbc.RA118.002552 | Google Scholar 29626096 PMC5971461

[B19] Lau N. Pluth M. D. (2019). Reactive Sulfur Species (RSS): Persulfides, Polysulfides, Potential, and Problems. Curr. Opin. Chem. Biol. 49, 1–8. 10.1016/j.cbpa.2018.08.012 PubMed Abstract | 10.1016/j.cbpa.2018.08.012 | Google Scholar 30243097

[B20] Leung C. W. Hong Y. Chen S. Zhao E. Lam J. W. Tang B. Z. (2013). A Photostable AIE Luminogen for Specific Mitochondrial Imaging and Tracking. J. Am. Chem. Soc. 135 (1), 62–65. 10.1021/ja310324q PubMed Abstract | 10.1021/ja310324q | Google Scholar 23244346

[B21] Qin J. Peng Z. Li B. Ye K. Zhang Y. Yuan F. (2015). Gold Nanorods as a Theranostic Platform for *In Vitro* and *In Vivo* Imaging and Photothermal Therapy of Inflammatory Macrophages. Nanoscale 7 (33), 13991–14001. 10.1039/c5nr02521d PubMed Abstract | 10.1039/c5nr02521d | Google Scholar 26228112

[B22] Rezaeian A. H. Wei W. Inuzuka H. (2022). The Regulation of Neuronal Autophagy and Cell Survival by MCL1 in Alzheimer's Disease. Acta Mater. Med. 1 (1), 42–55. 10.15212/amm-2021-0002 PubMed Abstract | 10.15212/amm-2021-0002 | Google Scholar 35233562 PMC8883233

[B23] Sasaki S. (2010). Determination of Altered Mitochondria Ultrastructure by Electron Microscopy. Methods Mol. Biol. 648, 279–290. 10.1007/978-1-60761-756-3_19 PubMed Abstract | 10.1007/978-1-60761-756-3_19 | Google Scholar 20700720

[B24] Schapira A. H. (2006). Mitochondrial Disease. Lancet 368, 70–82. 10.1016/S0140-6736(06)68970-8 PubMed Abstract | 10.1016/S0140-6736(06)68970-8 | Google Scholar 16815381

[B25] Senyilmaz D. Virtue S. Xu X. Tan C. Y. Griffin J. L. Miller A. K. (2015). Regulation of Mitochondrial Morphology and Function by Stearoylation of TFR1. Nature 525 (7567), 124–128. 10.1038/nature14601 PubMed Abstract | 10.1038/nature14601 | Google Scholar 26214738 PMC4561519

[B26] Shao X. Chen Q. Hu L. Tian Z. Liu L. Liu F. (2020). Super-resolution Quantification of Nanoscale Damage to Mitochondria in Live Cells. Nano Res. 13, 2149–2155. 10.1007/s12274-020-2822-9 10.1007/s12274-020-2822-9 | Google Scholar

[B27] Shi B. Jie K. Zhou Y. Zhou J. Xia D. Huang F. (2016). Nanoparticles with Near-Infrared Emission Enhanced by Pillararene-Based Molecular Recognition in Water. J. Am. Chem. Soc. 138 (1), 80–83. 10.1021/jacs.5b11676 PubMed Abstract | 10.1021/jacs.5b11676 | Google Scholar 26699758

[B28] Shi D. Liu W. Wang G. Guo Y. Li J. (2022). Small-molecule Fluorescence-Based Probes for Aging Diagnosis. Acta Materia. Med. 1 (1), 4–23. 10.15212/AMM-2021-0004 10.15212/AMM-2021-0004 | Google Scholar

[B29] Tabassum R. Jeong N. Y. (2019). Potential for Therapeutic Use of Hydrogen Sulfide in Oxidative Stress-Induced Neurodegenerative Diseases. Int. J. Med. Sci. 16 (10), 1386–1396. 10.7150/ijms.36516 PubMed Abstract | 10.7150/ijms.36516 | Google Scholar 31692944 PMC6818192

[B30] Vinten-Johansen J. (2020). Commentary: Mitochondria Are More Than Just the Cells' Powerhouse. J. Thorac. Cardiovasc. Surg. 160 (2), e33–e34. 10.1016/j.jtcvs.2019.07.029 PubMed Abstract | 10.1016/j.jtcvs.2019.07.029 | Google Scholar 31444067

[B31] Wiemerslage L. Lee D. (2016). Quantification of Mitochondrial Morphology in Neurites of Dopaminergic Neurons Using Multiple Parameters. J. Neurosci. Methods 262, 56–65. 10.1016/j.jneumeth.2016.01.008 PubMed Abstract | 10.1016/j.jneumeth.2016.01.008 | Google Scholar 26777473 PMC4775301

[B32] Yan Y. H. He X. Y. Miao J. Y. Zhao B. X. (2019). A Near-Infrared and Mitochondria-Targeted Fluorescence Probe for Ratiometric Monitoring of Sulfur Dioxide Derivatives in Living Cells. J. Mater. Chem. B. 7 (42), 6585–6591. 10.1039/c9tb01686d PubMed Abstract | 10.1039/c9tb01686d | Google Scholar 31589220

[B33] Yousif L. F. Stewart K. M. Kelley S. O. (2009). Targeting Mitochondria with Organelle-specific Compounds: Strategies and Applications. Chembiochem 10, 1939–1950. 10.1002/cbic.200900185 PubMed Abstract | 10.1002/cbic.200900185 | Google Scholar 19637148

[B34] Yu T. Robotham J. L. Yoon Y. (2006). Increased Production of Reactive Oxygen Species in Hyperglycemic Conditions Requires Dynamic Change of Mitochondrial Morphology. Proc. Natl. Acad. Sci. U S A. 103 (8), 2653–2658. 10.1073/pnas.0511154103 PubMed Abstract | 10.1073/pnas.0511154103 | Google Scholar 16477035 PMC1413838

[B35] Zhao E. Deng H. Chen S. Hong Y. Leung C. W. Lam J. W. (2014). A Dual Functional AEE Fluorogen as a Mitochondrial-specific Bioprobe and an Effective Photosensitizer for Photodynamic Therapy. Chem. Commun. (Camb) 50 (92), 14451–14454. 10.1039/c4cc07128j PubMed Abstract | 10.1039/c4cc07128j | Google Scholar 25302466

[B36] Zhao N. Chen S. Hong Y. Tang B. Z. (2015). A Red Emitting Mitochondria-Targeted AIE Probe as an Indicator for Membrane Potential and Mouse Sperm Activity. Chem. Commun. (Camb) 51 (71), 13599–13602. 10.1039/c5cc04731e PubMed Abstract | 10.1039/c5cc04731e | Google Scholar 26264419

[B37] Zhao N. Li M. Yan Y. Lam J. W. Y. Zhang Y. L. Zhao Y. S. (2013). A Tetraphenylethene-Substituted Pyridinium Salt with Multiple Functionalities: Synthesis, Stimuli-Responsive Emission, Optical Waveguide and Specific Mitochondrion Imaging. J. Mater. Chem. C 1, 4640. 10.1039/c3tc30759j 10.1039/c3tc30759j | Google Scholar

