# Supplementary material for: A Dual-Labeling Probe for Super-Resolution Imaging to Detect Mitochondrial Reactive Sulfur Species in Live Cells
Source: Front Pharmacol. 2022 Jun 1;13:871059. doi: 10.3389/fphar.2022.871059 (PMC9198575; doi:10.3389/fphar.2022.871059)
Supplement: Supplementary file 1 [file DataSheet1.PDF]

Supporting information

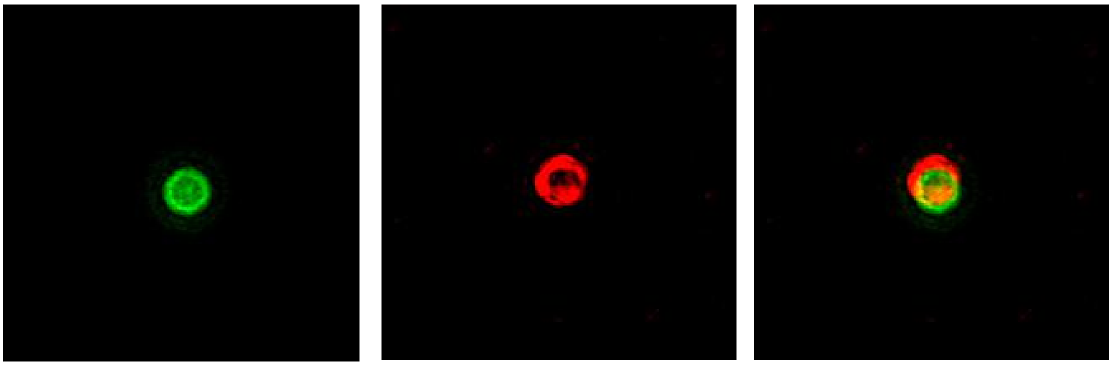

FigureS1. Image of **CPE** and lecithin under SIM

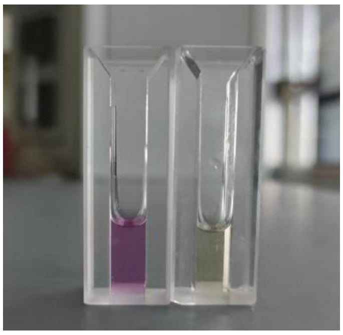

FigureS2. Color change of **CPE** with Na_2SO_3 in or not

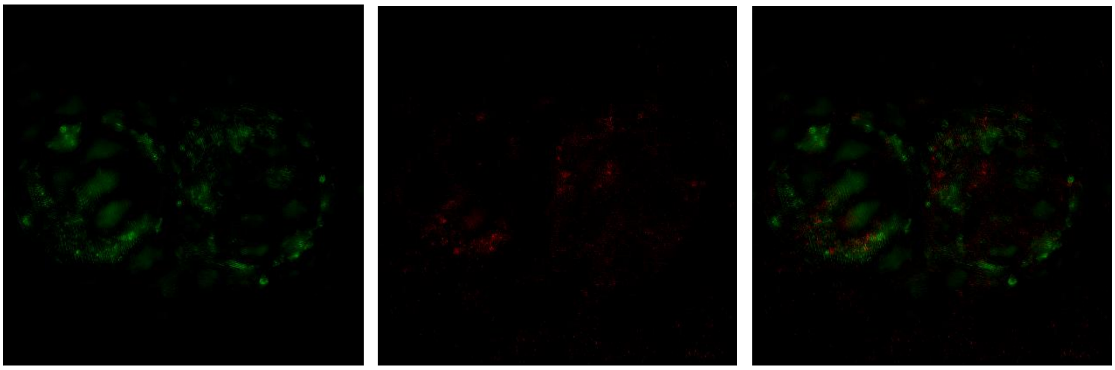

FigureS3. Hela cells incubated in 4℃

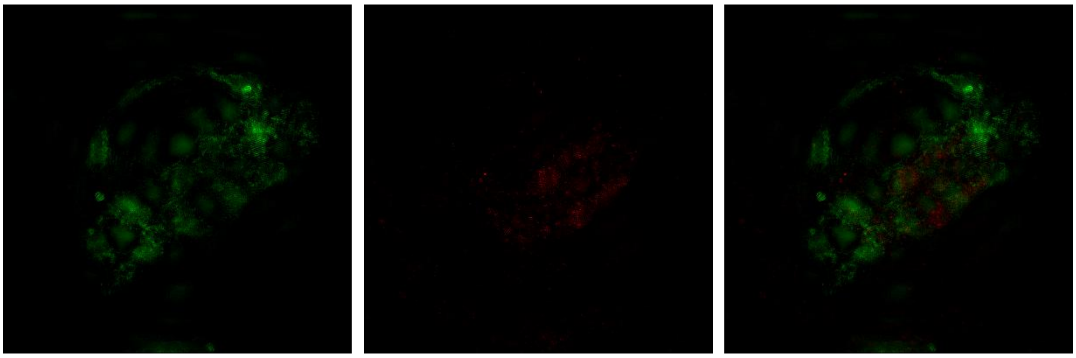

FigureS4. Hela cells treated with NH_4Cl

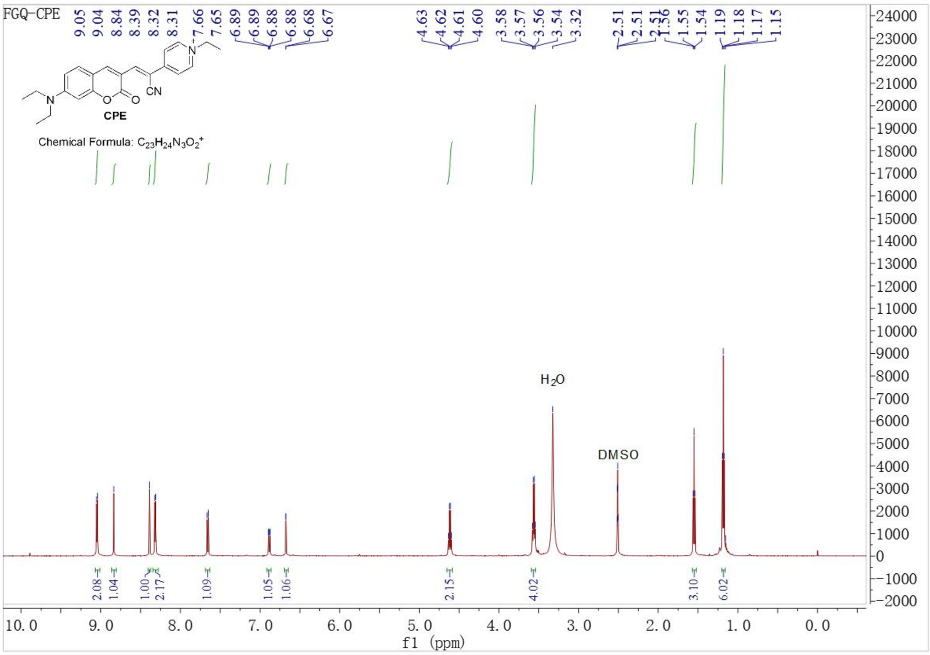

Figure S5. ^{1}H NMR spectrum of **CPE**

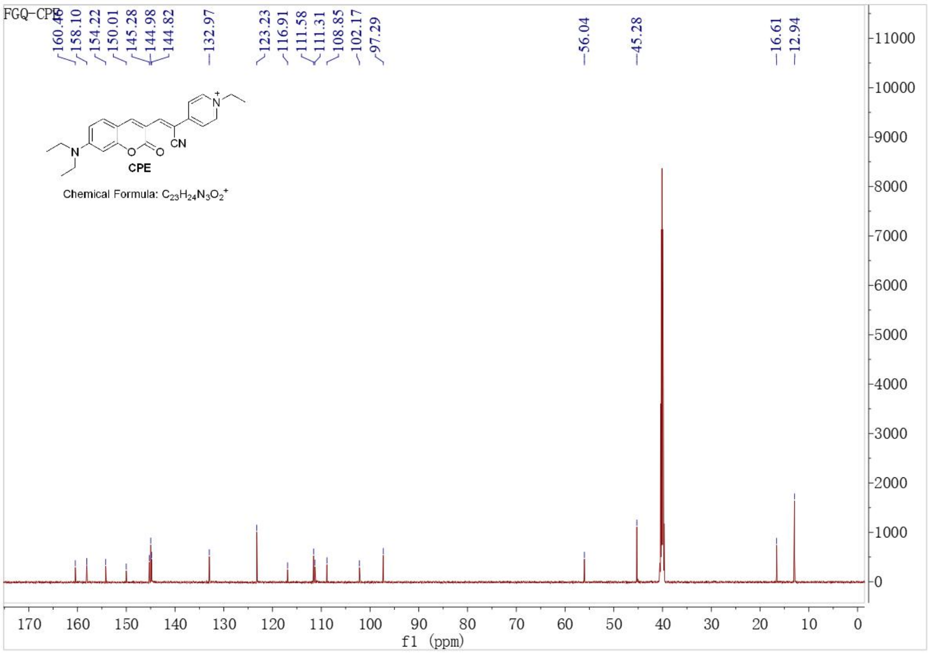

FigureS6. ^{13}C NMR spectrum of **CPE**

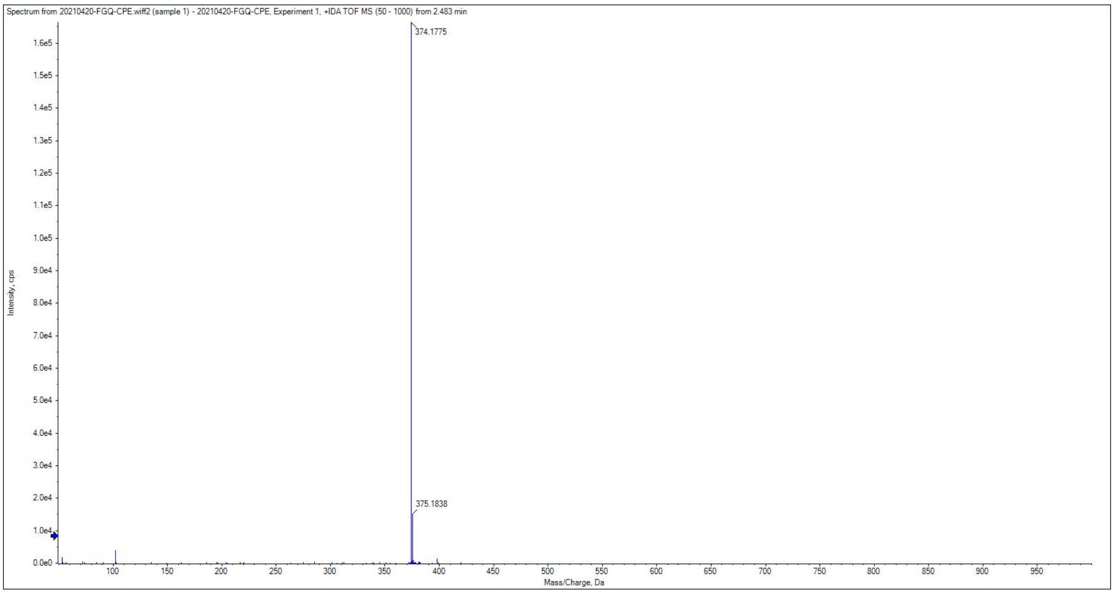

FigureS7. HRMS spectrum of **CPE**.
